# From K_6_[Re_6−x_Mo_x_S_8_(CN)_5_] Solid Solution to Individual Cluster Complexes: Separation and Investigation of [Re_4_Mo_2_S_8_(CN)_6_]^n−^ and [Re_3_Mo_3_S_8_(CN)_6_]^n−^ Heterometallic Clusters

**DOI:** 10.3390/molecules28155875

**Published:** 2023-08-04

**Authors:** Tatiana I. Lappi, Yakov M. Gayfulin, Adèle Renaud, Carmelo Prestipino, Pierric Lemoine, Vadim V. Yanshole, Viktoria K. Muravieva, Stéphane Cordier, Nikolai G. Naumov

**Affiliations:** 1 Nikolaev Institute of Inorganic Chemistry SB RAS, 3, Acad. Lavrentiev Ave., 630090 Novosibirsk, Russia; tanya.lappi2@gmail.com (T.I.L.); gayfulin@niic.nsc.ru (Y.M.G.); muravyeva@niic.nsc.ru (V.K.M.); 2 UFR Sciences et Propriétés de la Matière, Université de Rennes, CNRS, Institut des Sciences Chimiques de Rennes (ISCR) UMR 6226, F-35000 Rennes, France; adele.renaud@univ-rennes1.fr (A.R.); carmelo.prestipino@univ-rennes1.fr (C.P.); stephane.cordier@univ-rennes1.fr (S.C.); 3 Institut Jean Lamour, UMR 7198 CNRS, Universite de Lorraine, F-54011 Nancy, France; pierric.lemoine@univ-lorraine.fr; 4 International Tomography Center SB RAS, 3A, Institutskaya Str., 630090 Novosibirsk, Russia; vadim.yanshole@tomo.nsc.ru

**Keywords:** metal cluster, heterometallic, crystal structure, electronic structure, DFT calculations, EXAFS, electrochemistry

## Abstract

A series of new cluster compounds with {Re_4_Mo_2_S_8_} and {Re_3_Mo_3_S_8_} cores has been obtained and investigated. The clusters with different Re/Mo ratios were isolated as individual compounds, which made it possible to study their spectroscopic and electrochemical properties. The geometry of the new clusters was studied using a combination of X-ray diffraction analysis, XAS and quantum chemical DFT calculations. It was shown that the properties of the new clusters, such as the number and position of electrochemical transitions, electronic structure and change in geometry with a change in charge, are similar to the properties of clusters based on the {Re_4_Mo_2_Se_8_} and {Re_3_Mo_3_Se_8_} cores described earlier.

## 1. Introduction

Chemical and physical properties, as well as the application potential of octahedral cluster complexes based on {M_6_(µ_3_-Q)_8_} cores (M = Re, Q = S, Se, or Te; M = Mo or W, Q = Cl, Br, or I), attract great interest and have been intensively studied over the past few decades [1,2,3,4]. Within the {M_6_Q_8_} core, the octahedral M_6_ metal cluster is surrounded by eight inner ligands Q, which are bound to the faces of the octahedron. Each metal atom is coordinated by one apical ligand L, forming a [{M_6_Q_8_}L_6_]^n^ cluster unit. The chemical composition of the {M_6_Q_8_} cluster core and, in particular, the nature of metal atoms largely determine the physical properties of the cluster-based compounds, such as their redox properties, phosphorescence, or solar light-harvesting performances [5,6,7,8,9]. The synthesis of heterometallic clusters based on M_6−x_M′_x_ clusters with a controlled M/M′ ratio may lead to a smooth and predictable change in the properties of clusters. This is a promising approach for the design of cluster-based materials with predefined functionality. However, this approach may be quite difficult to realize since the octahedral cluster cores are usually formed during high-temperature synthesis and are chemically inert to the substitution of metal atoms at lower temperatures.

Solid-state Chevrel phases with the composition Mo_2_Re_4_Q_8_ (Q = S, Se) were the first published examples of compounds based on heterometallic octahedral clusters. They were obtained by the high-temperature reaction between stoichiometric quantities of simple substances [10]. Similar experimental protocols were used to prepare other {Mo_6−x_M′_x_Q_8_} heterometallic polymeric cluster-based phases (M′ = Re, Q = Te, x = 2; M′ = Ru, Q = Te, 1.5 ≤ x ≤ 2, Q = Se, x = 2; M′ = Rh, Q = Te, x = 0.5, 1.33) [11,12,13,14,15,16], as well as Cs_3_Re_5_OsS_11_ [17]. Note that, for Mo-M′-Q systems, well-defined compositions with integer x values (Mo_4_Ru_2_Te_8_; Mo_4_Ru_2_Se_8_; Mo_5_Ru_1_Te_8_) could be prepared, as well as compositions with non-integer x values (Mo_4.5_Ru_1.5_Te_8_; Mo_1.5_Re_4.5_Se_8_; Mo_4.66_Rh_1.33_Te_8_). This indicates the coexistence of at least two cluster cores with different Mo/M′ ratios in the form of a solid solution. The separation of the different cluster cores {Mo_6−x_M′_x_Q_8_} for each integer value of x from the solid-state precursor requires a combination of solid-state and solution chemistry techniques in order to form [Mo_6−x_M′_x_Q_8_L_6_] soluble cluster units.

Soluble heterometallic octahedral clusters were obtained for the following metal combinations: rhenium/osmium [18,19,20], molybdenum/niobium [21] and rhenium/molybdenum [22,23]. All the compounds were obtained by high-temperature reactions between simple substances and binary precursors. Clusters with {Re_6−x_Os_x_Se_8_} (x = 1–3) and {Mo_5_NbI_8_} cores can be isolated as individual species directly from the reaction mixtures. Meanwhile, clusters with {Re_6−x_Mo_x_Q_8_} (Q = S, Se; x = 1–3) cores were formed as apparent single-phase solid solutions but contained simultaneously several cluster cores with the same charges and different values of x. The separation of the different cluster cores proved to be a non-trivial task since they have similar geometric parameters and charges and do not separate chromatographically. Very recently, the separation and detailed investigation of [Re_6−x_Mo_x_Se_8_(CN)_6_]^n−^ cluster units (x = 1–3) were reported [24,25]. It was shown that the variation in the Re/Mo ratio led to drastic changes in the redox and spectroscopic properties of the clusters, which may allow for the obtaining of sensor and electrochromic materials based on these functionalized species. The aforementioned complicated nature of solid-state phases based on {Re_6−x_Mo_x_Se_8_} units led us to re-investigate the “sulfide” solid-state phases formed as a result of the reactions between ReS_2_, MoS_2_ and KCN and reported initially as constant composition phase K_6_[Re_3_Mo_3_S_8_(CN)_5_] [23]. Recently, it was found that the synthesis of that chemical composition at different temperatures led to the formation of K_6_[Re_6−x_Mo_x_S_8_(CN)_5_] solid solutions with 2.75 < x < 3.63 [26]. Here we report on the separation procedures that allowed us to obtain salts of [Re_4_Mo_2_S_8_(CN)_6_]^n−^ and [Re_3_Mo_3_S_8_(CN)_6_]^n−^ clusters as individual species. Moreover, we report on the spectroscopic properties and crystal structure investigations and compare them with the properties of the corresponding {Re_4_Mo_2_Se_8_} and {Re_3_Mo_3_Se_8_} cluster cores.

## 2. Results and Discussion

### 2.1. Synthesis

The reaction of ReS_2_ and MoS_2_ with KCN at 700 °C for 7 days led to the formation of the polymeric compound K_6_[Re_3.16_Mo_2.84_S_8_(CN)_5_] (**1**) [26]. The crystal structure of this compound was described earlier [15]. It is based on cluster units polymerized by means of bridging cyanide groups in *trans*-position to form chains. It should be noted that the ReS_2_ and MoS_2_ precursors used in the synthesis of **1** were obtained at 1000 °C. The preparation of metal sulfides at lower temperatures may lead to the formation of polymer K_6_[Re_6-x_Mo_x_S_8_(CN)_5_] with different Re/Mo ratio. 

**Figure 1:**
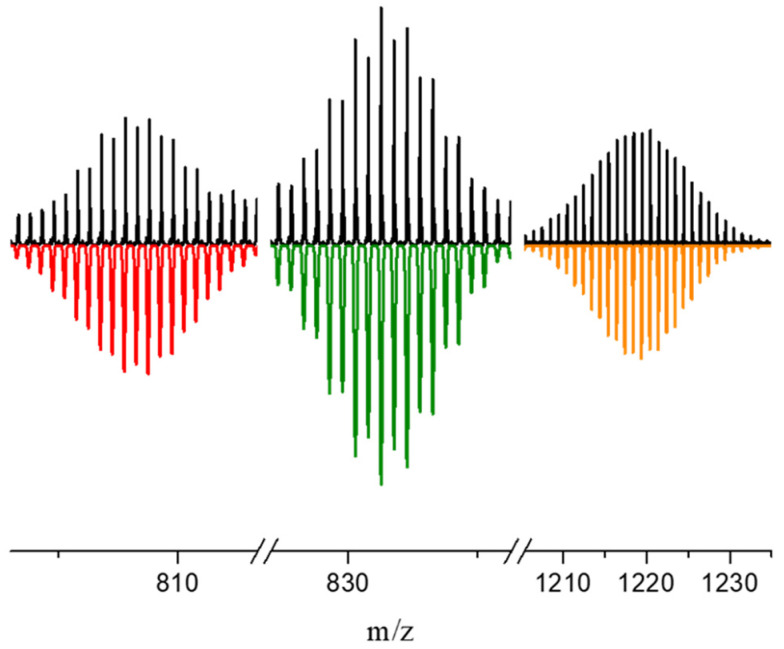
Observed (**top**) vs. calculated (**bottom**) isotopic distribution peak sets corresponding to the cluster anionic adducts (Ph_4_P)[Re_3_Mo_3_S_8_(CN)_6_]·(H_2_O)^2−^ (*m*/*z*_calcd_ = 808.76 Da), (Ph_4_P)[Re_4_Mo_2_S_8_(CN)_5_]^2−^ (*m*/*z*_calcd_ = 831.28 Da) and H_2_[Re_2_Mo_4_S_8_(CN)_4_]·(H_2_O)(CH_3_CN)^2−^ (*m*/*z*_calcd_ = 1220.40 Da) in the CH_3_CN solution of compound **3**.

**Scheme 1:**
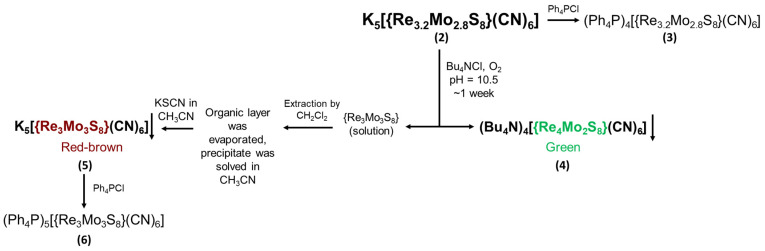
The scheme of procedure for separating individual compounds containing {Re_3_Mo_3_S_8_} and {Re_4_Mo_2_S_8_} cluster cores.

Compound **1** is insoluble in deaerated water. The treatment of the reaction products with boiling Ar-saturated H_2_O led to the dissolution of the KCN excess. The interaction of **1** with O_2_ in an aerated KCN solution resulted in depolymerization accompanied by two-electron oxidation of the cluster core and the formation of the discrete cluster anion [Re_3.2_Mo_2.8_S_8_(CN)_6_]^5−^, which was isolated and crystallized as potassium salt **2**. Note that the Re/Mo ratio in compound **2** was the same as in compound **1**, confirming the co-crystallization of several clusters with different cores in the reaction mixture. To determine the composition of the mixture of clusters, compound **2** was converted to (Ph_4_P)^+^ salt by mixing the aqueous solutions of **2** and Ph_4_PCl. The precipitate formed had the composition (Ph_4_P)_4_[Re_3.2_Mo_2.8_S_8_(CN)_6_] (**3**) (Scheme 1). The decoloration of the solution indicates the quantitative precipitation of the cluster products. Based on the results of the mass spectrometric analysis of a solution of this salt in CH_3_CN, it was demonstrated that compound **3** contains cluster units based on three different cluster cores, namely, [Re_4_Mo_2_S_8_(CN)_6_]^n−^, [Re_3_Mo_3_S_8_(CN)_6_]^n−^ and [Re_2_Mo_4_S_8_(CN)_6_]^n−^ (Figure 1).

To separate clusters with different Re/Mo ratios, an excess of Bu_4_NCl was added to the solution of compound **2** in H_2_O (Scheme 1). It was found that this reaction is pH-dependent. In the case of {Re_4_Mo_2_S_8_}, the addition of KOH can promote oxidation. Using a KOH solution, the pH value of the reaction mixture was adjusted to 10.5. The reaction mixture was left in a glass with air access for one week. As a result, a green crystalline precipitate of compound (Bu_4_N)_4_[Re_4_Mo_2_S_8_(CN)_6_] (**4**) was formed.

**Figure 2:**
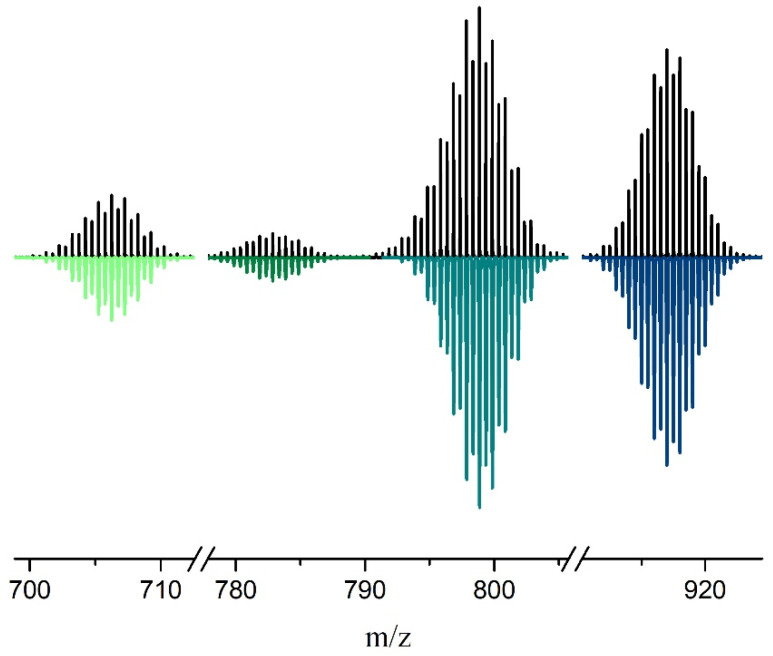
Observed (**top**) vs. calculated (**bottom**) isotopic distribution peak sets corresponding to the cluster adducts H_4_[Re_4_Mo_2_S_8_(CN)_6_]·(H_2_O)(CH_3_CN)^2−^ (706.24 Da), (Bu_4_N)[Re_4_Mo_2_S_8_(CN)_5_]^2−^ (782.85 Da), (Bu_4_N)[Re_4_Mo_2_S_8_(CN)_4_(OH)]·(CH_3_CN)^2−^ (798.86 Da) and (Bu_4_N)_2_[Re_4_Mo_2_S_8_(CN)_6_]^2−^ (916.99 Da) in the ESI mass spectrum of the CH_3_CN solution of compound **4**.

**Figure 3:**
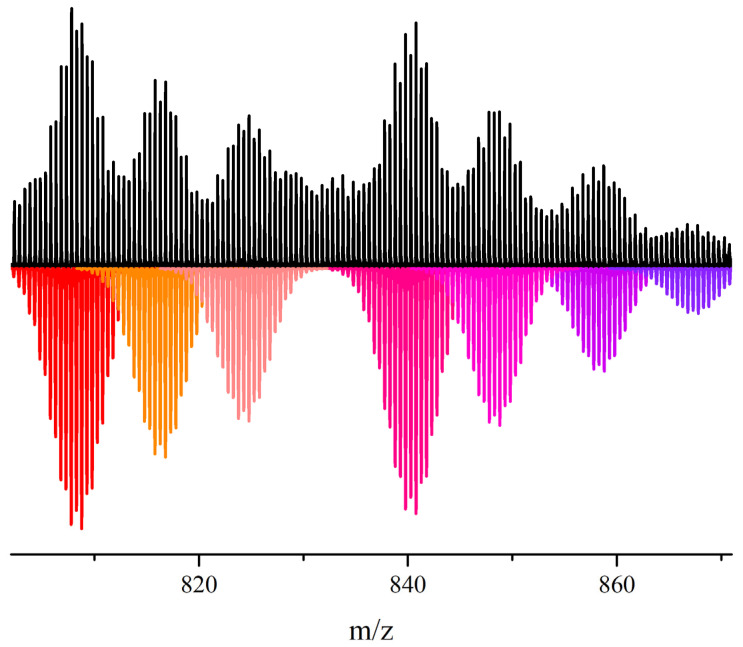
Observed (**top**) vs. calculated (**bottom**) isotopic distribution peak sets corresponding to the cluster adducts (Ph_4_P)H_3_[Re_3_Mo_3_S_8_(CN)_5_]·(CH_3_CN)^2−^ (808.27 Da), (Ph_4_P)H_4_[Re_3_Mo_3_S_8_(CN)_4_]·(CH_3_CN)_2_^2−^ (816.29 Da), (Ph_4_P)H_2_[Re_3_Mo_3_S_8_(CN)_4_]·(CH_3_CN)_2_(H_2_O)^2−^ (824.29 Da), (Ph_4_P)[Re_3_Mo_3_S_8_(CN)_6_]·(CH_3_CN)_2_^2−^ (840.28 Da), (Ph_4_P)H[Re_3_Mo_3_S_8_(CN)_5_]·(CH_3_CN)_3_^2−^ (848.29 Da), (Ph_4_P)[Re_3_Mo_3_S_8_(CN)_6_]·(CH_3_CN)_2_(H_2_O)_2_^2−^ (858.29 Da) and (Ph_4_P)[Re_3_Mo_3_S_8_(CN)_6_]·(CH_3_CN)_2_(H_2_O)_3_^2−^ (867.29 Da) in the ESI mass spectrum of the CH_3_CN solution of compound **6**.

One can see that during the reaction, one-electron oxidation of the [Re_4_Mo_2_S_8_(CN)_6_]^5−^ cluster (23 cluster skeletal electrons—CSEs, Table 1) to [Re_4_Mo_2_S_8_(CN)_6_]^4−^ (22 CSEs) occurred, causing the precipitation of salt **4**, which is insoluble in H_2_O. Further holding of the filtrate solution in air did not lead to the precipitation of other products; therefore, we can assume that quantitative separation of the cluster with {Re_4_Mo_2_S_8_} core took place. This was confirmed by the investigation of the solution of compound **4** by mass spectrometry. In the mass spectrum (Figure 2), all intense signals correspond to adducts of the [Re_4_Mo_2_S_8_(CN)_6_]^n−^ cluster. After the isolation of compound **4**, the aqueous reaction mixture remained colored, indicating the presence of other cluster compounds. This colored species was successfully extracted with CH_2_Cl_2_, then the organic layer was separated, evaporated to dryness in air and dissolved in CH_3_CN. A solution of KSCN in CH_3_CN was added, causing the quick precipitation of brown powder of K_5_[Re_3_Mo_3_S_8_(CN)_6_] (**5**) (Scheme 1). The aqueous solution of compound **5** was mixed with the aqueous solution of Ph_4_PCl, causing the immediate precipitation of compound **6**. As in the case of compound **3**, compound **6** is soluble in CH_3_CN, allowing it to be examined by ESI-MS. The main isotopic distribution peak sets found match well the {Re_3_Mo_3_S_8_}-based cluster adducts (Figure 3). Therefore, the separation and selective isolation of clusters with the {Re_4_Mo_2_S_8_} and {Re_3_Mo_3_S_8_} cores were carried out.

### 2.2. Crystal Structures

**Figure 4:**
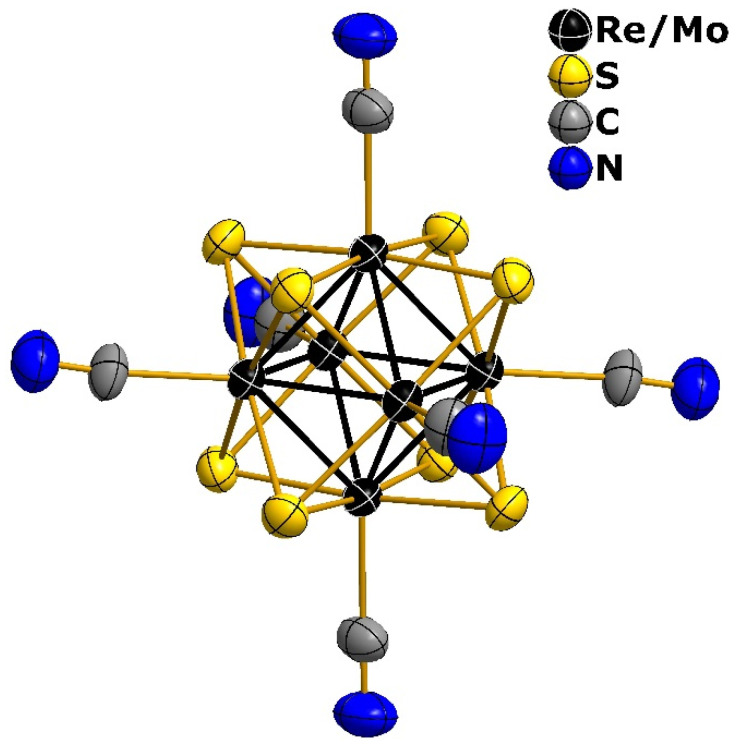
Geometry of the [Re_4_Mo_2_S_8_(CN)_6_]^4−^ cluster anion in the structure of compound **4**. Thermal ellipsoids of 50% probability are given.

Compounds (Bu_4_N)_4_[Re_4_Mo_2_S_8_(CN)_6_]·2H_2_O (**4**) and K_5_[Re_3_Mo_3_S_8_(CN)_6_]·8H_2_O (**5**) were investigated by single-crystal X-ray diffraction. The crystal structures of these compounds are based on cluster anions [Re_4_Mo_2_S_8_(CN)_6_]^4−^ and [Re_3_Mo_3_S_8_(CN)_6_]^5−^, respectively, both having 22 CSEs. The anions exhibit the typical geometry of octahedral cluster complexes of the [M_6_Q_8_L_6_] type (Figure 4). The rhenium and molybdenum atoms in both structures are disordered over all positions of the metal atoms. The refinement of the site occupancies leads to chemical compositions Re_3.87(3)_Mo_2.13(3)_ and Re_2.94(12)_Mo_3.06(12)_, which are close to the integer values {Re_4_Mo_2_} and {Re_3_Mo_3_}. As XRD analysis provides only an average structure, it was impossible to establish the isomeric composition of the obtained clusters as well as the local Re–Re, Mo–Mo and Re–Mo interatomic distances. Nevertheless, an increase in the average interatomic M–M (2.615(1) Å and 2.623(2) Å) and M–Q (2.427(2) Å and 2.430(4) Å) distances correlated with a decrease in the Re/Mo ratio in the cluster core (compounds **4** and **5**, respectively, Table 2). This correlation was found earlier for selenide heterometallic clusters [24]. M–Q and M–M are shorter for sulfide heterometallic clusters than for selenide ones, in accordance with the difference in the atomic radii of sulfur and selenium.

### 2.3. Electrochemical Properties

**Figure 5:**
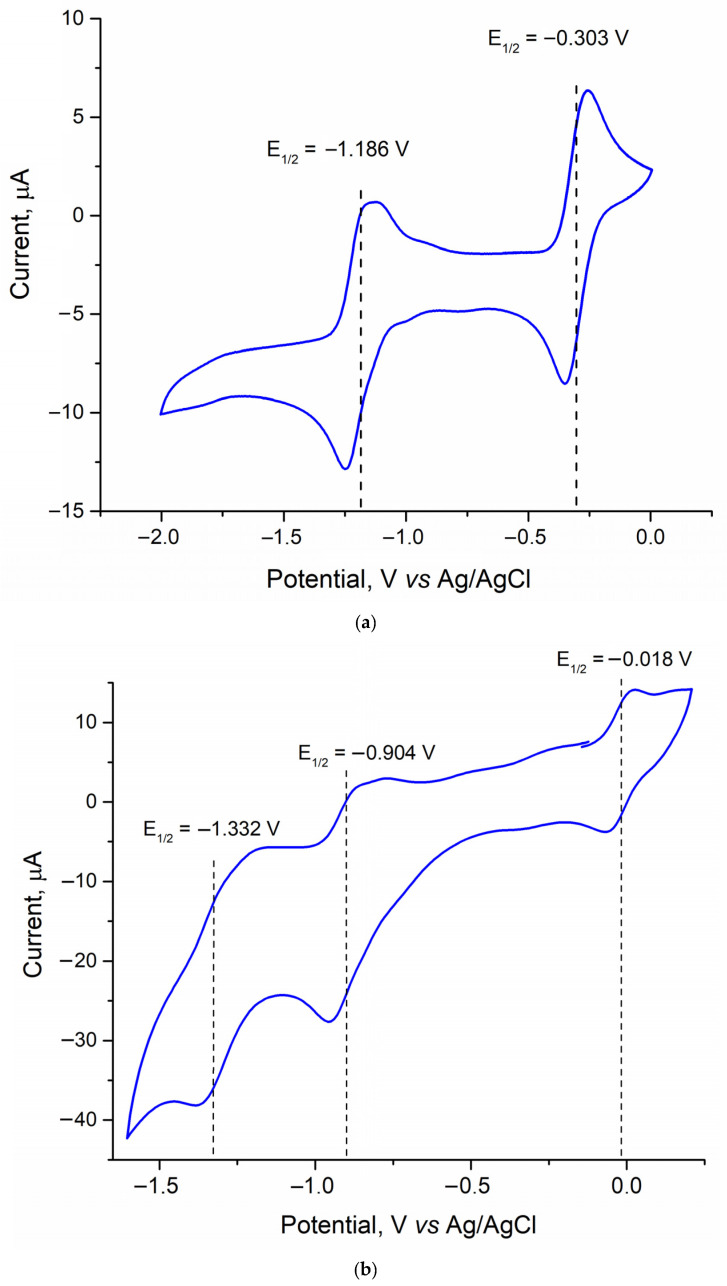
Cyclic voltammograms of compound **3** in acetonitrile solution (**a**) and compound **5** in acetone solution (**b**) vs. Ag/AgCl reference electrode. Scan rate was 100 mV·s^−1^.

The separation of the octahedral heterometallic clusters using the differences in their redox properties and solubility was first described for the clusters with {Re_4_Mo_2_Se_8_} and {Re_3_Mo_3_Se_8_} cores [24]. In that case, the process of precipitation of the (Bu_4_N)_4_[Re_4_Mo_2_Se_8_(CN)_6_] salt took only about one hour to be completed, as opposed to the one week needed to obtain compound **4**. Attempts to speed up the precipitation of compound **4** by heating the solution and adding excess Bu_4_N^+^ or other alkylammonium cations have not been successful. In the CH_3_CN solution, the [Re_4_Mo_2_S_8_(CN)_6_]^4−^ cluster shows two quasi-reversible transitions with E_1/2_ = −0.303 V and −1.186 V vs. Ag/AgCl (Figure 5a). These transitions correspond to the reduction of [Re_4_Mo_2_S_8_(CN)_6_]^4−^ (22 CSEs) in [Re_4_Mo_2_S_8_(CN)_6_]^5−^ (23 CSEs) and then in [Re_4_Mo_2_S_8_(CN)_6_]^6−^ (24 CSEs, Table 1), respectively. In comparison with the [Re_4_Mo_2_S_8_(CN)_6_]^n−^ clusters, the potentials of the corresponding transitions are shifted by 0.173 and 0.108 V to the anodic region (Table 3). This shift correlates well with the known electrochemical data for homometallic octahedral clusters of rhenium [Re_6_Q_8_(CN)_6_]^4−/3−^, where a successive cathodic shift of the oxidation potentials occurs upon changing the nature of the inner ligands from S to Se and Te [27,28]. Salt **4** can be transformed to the water-soluble salt K_5_[Re_4_Mo_2_S_8_(CN)_6_] by adding a solution of KSCN in CH_3_CN to the solution of **4** in CH_3_CN. The cyclic voltammetry curve for the K_5_[Re_4_Mo_2_S_8_(CN)_6_] salt in aqueous solution is characterized by one reversible redox transition with E_1/2_ at 0.073 V vs. Ag/AgCl (Figure S7). This transition corresponds to the oxidation of [Re_4_Mo_2_S_8_(CN)_6_]^5−^ to obtain [Re_4_Mo_2_S_8_(CN)_6_]^4−^ (22 to 23 CSEs).

The cyclic voltammogram of the [Re_3_Mo_3_S_8_(CN)_6_]^5−^ anion (22 CSEs, compound **5**) in acetone contains three quasi-reversible redox waves (Figure 5b). One of them corresponds to the oxidation process, while the two remaining waves correspond to reduction processes. Therefore, four charge states for [Re_3_Mo_3_S_8_(CN)_6_]^n−^ anions are electrochemically evidenced, namely, [Re_3_Mo_3_S_8_(CN)_6_]^4−/5−/6−/7−^, containing 21 to 24 CSEs, respectively. The half-wave potentials of these processes are −0.018, −0.904 and −1.332 V, respectively, vs. Ag/AgCl. These values are close to the previously reported ones for [Re_3_Mo_3_Se_8_(CN)_6_]^4−/5−/6−/7−^ anions [24], with the exception of the 21/22 CSE transition for [Re_3_Mo_3_Q_8_(CN)_6_]^4−/5−^ anions (Table 3). One can notice that the nature of the inner chalcogenide ligand has much less impact on the electrochemical behavior of clusters with a higher content of molybdenum forming the cluster.

One may see that embedding molybdenum atoms instead of rhenium ones within the {Re_6_} metal core increases the number of electrochemically accessible redox transitions of the resulting compounds and shifts them to the negative potential region (Table 3. The [Re_6_S_8_(CN)_6_]^4−^ complex is characterized by one redox transition corresponding to a change in the number of CSEs from 24 to 23 at +0.60 V vs. Ag/AgCl [27]. In the same conditions, the [Re_4_Mo_2_S_8_(CN)_6_]^6−/5−^ redox transition (24 to 23 CSEs) displayed a potential of −1.186 V. The substitution of one more rhenium atom for molybdenum led to the shift of the 24-to-23-CSE transition ([Re_3_Mo_3_S_8_(CN)_6_]^7−/6−^ redox pair) to −1.332 V. Moreover, {Re_3_Mo_3_} heterometallic cluster complexes are accessible as stable compounds in highly oxidized forms up to 21 CSEs. Thus, the substitution of metals in the metal cores significantly affects the redox properties of cluster complexes, making it possible to vary the optical and electronic properties of cluster-based materials by applying voltage.

### 2.4. Electronic Structure

**Figure 6:**
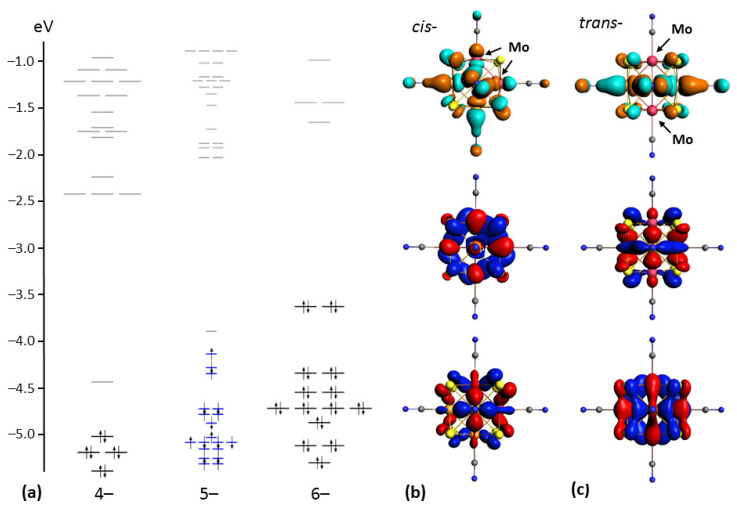
Energy level diagrams of *trans*-isomer of [Re_4_Mo_2_S_8_(CN)_6_]^n−^ anions (n = 4–6 from left to right, (**a**)). Typical view of HOMO–1, HOMO and LUMO for *cis*-[Re_4_Mo_2_S_8_(CN)_6_]^6−^ anion (**b**). Typical view of HOMO–1, HOMO and LUMO for *trans*-[Re_4_Mo_2_S_8_(CN)_6_]^6−^ anion (**c**). Isosurface isovalues are ±0.03 a.u.

**Figure 7:**
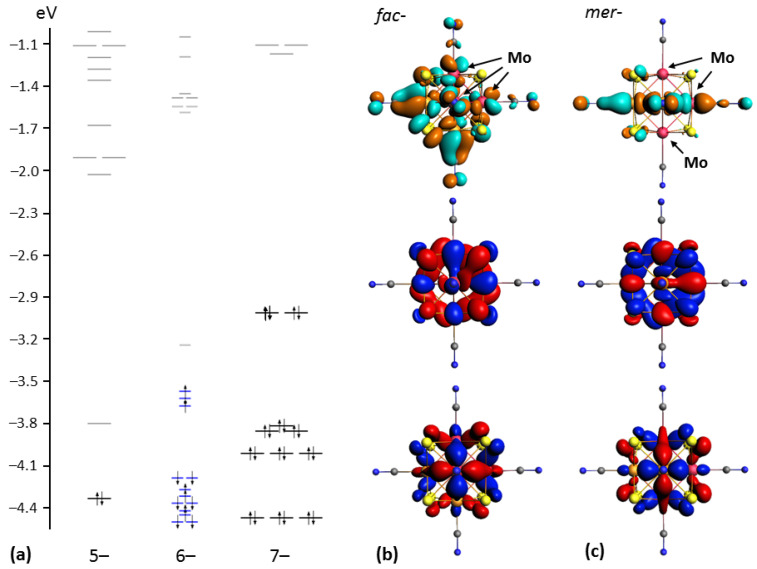
Energy level diagrams of *fac*-isomer of [Re_3_Mo_3_S_8_(CN)_6_]^n−^ anions (n = 5–7 from left to right, (**a**)). Typical view of HOMO–1, HOMO and LUMO for *fac*-[Re_3_Mo_3_S_8_(CN)_6_]^7−^ anion (**b**). Typical view of HOMO–1, HOMO and LUMO for *mer*-[Re_3_Mo_3_S_8_(CN)_6_]^7−^ anion (**c**). Isosurface isovalues are ±0.03 a.u.

In order to analyze the geometry and electronic structure of novel heterometallic cores {Re_4_Mo_2_S_8_} and {Re_3_Mo_3_S_8_}, DFT calculations were performed for the *cis*- and *trans*-isomers of [Re_4_Mo_2_S_8_(CN)_6_]^n−^ anions (n = 4–6, Figure 6), as well as for the *fac*- and *mer*-isomers of [Re_3_Mo_3_S_8_(CN)_6_]^n−^ anions (n = 5–7, Figure 7). The CSE count for these anions varied from 22 to 24. The formation energies of all 24-electron anions were similar and equal to −226.518, −226.639, −228.969 and −228.855 eV for *cis*-[Re_4_Mo_2_S_8_(CN)_6_]^6−^, *trans*-[Re_4_Mo_2_S_8_(CN)_6_]^6−^, *fac*-[Re_3_Mo_3_S_8_(CN)_6_]^7−^ and *mer*-[Re_3_Mo_3_S_8_(CN)_6_]^7−^, respectively. The structure of the molecular orbitals for electron-saturated heterometallic clusters shows that the two upper occupied orbitals (HOMO and HOMO-1) are separated from the lower orbitals by a relatively large energy gap. This was also mentioned for the clusters with {Re_3_Mo_3_Se_8_} and {Re_4_Mo_2_Se_8_} cores. The value of this energy gap is in the range of 0.67–0.80 eV. Frontier orbitals of clusters with 24 CSEs have bonding nature relative to metal–metal interactions below the Fermi energy level and anti-bonding characteristics above the Fermi energy level (LUMO, LUMO+1, etc.). HOMO and HOMO-1 are composed mostly of rhenium and molybdenum atomic orbitals, with some contribution of sulfur atomic orbitals (about 20%), while LUMO also has a notable contribution of C and N atomic orbitals. The HOMO–LUMO gaps are equal to about 2.2 eV for clusters with {Re_3_Mo_3_S_8_} cores and 2.0 eV for clusters with {Re_4_Mo_2_S_8_} cores.

The removal of electrons from HOMO causes a shift of HOMO–1 down in energy. The energy gap between SOMO and LUMO remains almost the same as the HOMO–LUMO gap for 24-electron anions. Further oxidation of the clusters causes a lowering of HOMO in energy. As a result, 22-electron clusters do not show a large gap between HOMO and the lower orbitals. On the contrary, the energy gap between HOMO and LUMO in 22-electron clusters (HOMO and HOMO–1 in 24-electron clusters) increases and reaches 0.5 eV. The gap between LUMO and LUMO+1 (HOMO–LUMO gap in 24-electron clusters) remains almost unchanged.

### 2.5. Geometry Optimization

**Figure 8:**
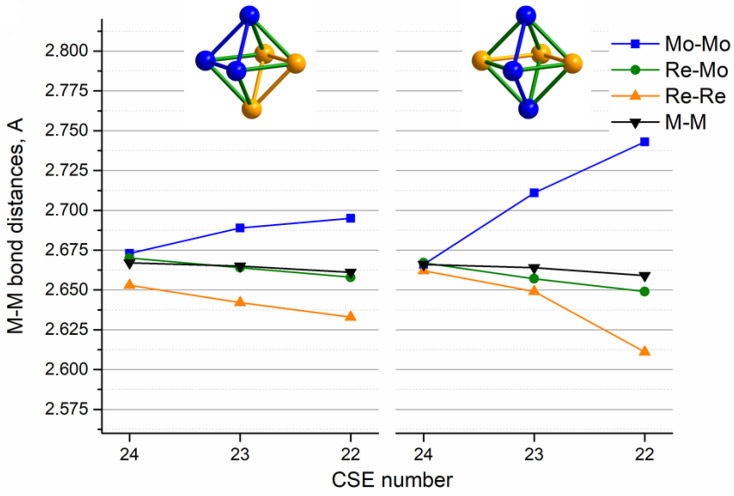
M–M and M–M′ bond distances (average Mo-Mo, Re-Mo, Re-Re and overall average M-M) of [Re_3_Mo_3_S_8_(CN)_6_]^n−^ (Å) according to optimized geometry from DFT calculations vs. CSE value of cluster anions.

According to the calculation data, the 24-electron metal core {Re_3_Mo_3_} is close to octahedron shape, especially for the *mer*-isomer and, to a lesser extent, for the *fac*-isomer, with little difference in M-M bond distances (Figure 8, 24 CSEs). The two-electron removal leads to a significant shortening of the Re-Re distances and an elongation of the Mo-Mo distances in the metal core in the case of both isomers (Figure 8, 22 CSEs). The distortion of the octahedron leads to *C_2v_* and *C_s_* metal cores for *mer-* and *fac-*isomers, respectively. The difference between the longest and shortest metal–metal distances for the 22-electron *mer-*isomer is 0.133 Å (for the *fac-*isomer, 0.182 Å).

**Figure 9:**
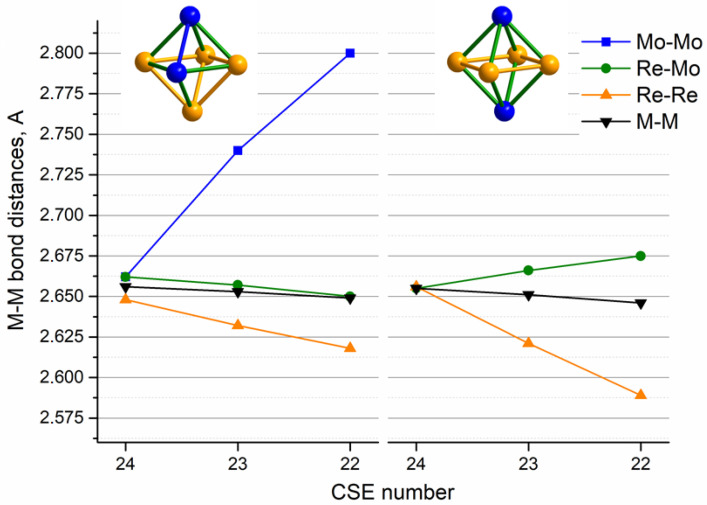
Core M-M bond distances (average Mo–Mo, Re–Mo, Re–Re and overall average M–M) of [Re_4_Mo_2_S_8_(CN)_6_]^n−^ (Å) according to optimized geometry from DFT calculations vs. CSE value of cluster anions.

A similar distortion character is observed for the {Re_4_Mo_2_} cluster, where two-electron removal also leads to the shortening of the Re–Re distances and the elongation of the Mo–Mo distances, giving *C_2v_* and *D_4h_* metal cores for *cis-* and *trans-*isomers, respectively (Figure 9).

The local coordination environment of rhenium and molybdenum atoms in the 22-electron {Re_3_Mo_3_} and {Re_4_Mo_2_} clusters was experimentally investigated by using the EXAFS method. It can be seen that, in both cases, the Re–Re distances are shorter than the average M–M distances, and the Mo–Mo distances are noticeably longer (Table 4 and Table 5, EXAFS column). It was shown that the interatomic M–M distances in the [Re_3_Mo_3_S_8_(CN)_6_]^5−^ and [Re_4_Mo_2_S_8_(CN)_6_]^4−^ cluster-based anionic units, determined theoretically by the method of quantum-chemical DFT calculations and experimentally from the EXAFS spectra, agree well with each other. Both approaches show that the {Re_3_Mo_3_} and {Re_4_Mo_2_} clusters in the cluster-based anionic units are strongly distorted in comparison with the experimental structural data obtained by X-ray diffraction. The resulting distortion cannot be determined from the structural data obtained by X-ray diffraction analysis since the symmetry of the cluster does not coincide with the symmetry of the structures, and the diffraction data contain the average positions of the metal atoms and, hence, the average interatomic distances. However, it was not possible to unambiguously determine by EXAFS the type of core isomerism from the obtained data due to the close values of the r-factor from fitting modeled data with different isomers (Tables S1–S4 in Supplementary Materials, r-factor values).

## 3. Experimental Section

### 3.1. Materials and Methods

All reagents and solvents were used as purchased. Elemental analysis was made on a EuroVector EA3000 analyzer (EuroVector, Pavia, Italy). Energy dispersive spectroscopy (EDS) was performed on a Hitachi TM3000 TableTop SEM (Hitachi, Ltd., Chiyoda City, Tokyo, Japan) with Bruker QUANTAX 70 EDS equipment (Bruker Corporation, Billerica, MA, USA). FT-IR spectra in KBr pellets were recorded on a Bruker Scimitar FTS 2000 spectrometer in the range 4000–375 cm^−1^. UV-Vis absorption measurements were performed in the wavelength range of 400–800 nm on an Agilent Cary 60 spectrophotometer (Agilent Technologies, Inc., Santa Clara, CA, USA). Electrospray ionization mass spectrometry was performed on a Bruker maXis 4G high-resolution ESI-q-TOF spectrometer. The mass spectra were recorded under the following conditions: registration of negative ions from *m*/*z* = 600 Da to 4000 Da, voltage +2500 V, pressure on the nebulizer of 0.8 bar, drying gas flow of 4 L/min and drying gas temperature of 180 °C. Cyclic voltammetry was carried out on an Electrochemical Instruments Elins P-20 × 8 voltammetry analyzer (Electrochemical Instruments, Chernogolovka, Russia) using a three-electrode scheme with glassy carbon working, Pt auxiliary and Ag/AgCl/3.5M KCl reference electrodes. Investigations were carried out for 2.5·10^−3^ M solution of cluster salts **3** and **6** in a 0.1 M solution of Bu_4_NClO_4_ in CH_3_CN or (CH_3_)_2_O, respectively, under an Ar atmosphere. The registered value of E_1/2_ for the Fc^0/+^ couple was 0.440 V in the same conditions. Powder X-ray diffraction (PXRD) data were collected using a Philips PW 1820/1710 diffractometer (Cu, Kα radiation, graphite monochromator, Si as external reference).

### 3.2. Synthesis of Compounds

ReS_2_ and MoS_2_ were synthesized by the reaction between stoichiometric amounts of corresponding metal and sulfur in evacuated silica ampoules at 1000 °C for 48 h. Phase purity of binary precursors was confirmed by PXRD.

*Preparation of K_6_[Re_3.2_Mo_2.8_S_8_(CN)_5_]* (**1**). Compound **1** was prepared using a technique derived from that previously described [15]. ReS_2_ (1.02 g, 4 mmol), MoS_2_ (0.65 g, 4 mmol) and KCN (1.68 g, 25 mmol) were mixed in a silica ampoule. The ampoule was evacuated and sealed. The reaction was carried out at 700 °C for 8 days, and then the ampoule was cooled down to room temperature in 12 h. The reaction mixture was stirred in Ar-saturated H_2_O (250 mL) under Ar gas flow at room temperature for 1 h to remove unreacted KCN and was filtered off. The resulting solid residue was washed on a glass filter with an EtOH/H_2_O mixture (7/1 vol., 2 portions of 20 mL) and EtOH (30 mL). The mixture containing octahedral black crystals of **1** with an admixture of unreacted metal sulfides was separated by sonication in EtOH with subsequent decantation, and then product **1** was dried in air. Yield: 1.30 g (66% based on total amount of metals). EDS: K:Re:Mo:S = 5.7:3.2:2.8:8.3. FT-IR (KBr, cm^−1^): 2088 (C≡N). Phase purity of the product was confirmed by PXRD. PXRD also revealed that compound **1** is isostructural to the K_6_[Re_3_Mo_3_S_8_(CN)_5_] phase reported previously [15].

*Preparation of K_5_[Re_3.2_Mo_2.8_S_8_(CN)_6_]* (**2**). Compound **1** (0.50 g, 0.33 mmol) and KCN (0.05 g, 0.77 mmol) were dissolved in 20 mL of H_2_O. The solution was evaporated on heating in a glass to a volume of about 5 mL and then 10 mL of EtOH was added, causing immediate precipitation of compound **2**. The precipitate was separated by centrifugation, washed with an EtOH/H_2_O mixture (7/1 vol., 20 mL) and EtOH (20 mL) and dried in air. Yield: 0.34 g (70%). EDS: K:Re:Mo:S = 4.8:3.1:2.9:8.0. FT-IR (KBr, cm^−1^): v = 2103 (C≡N); v = 3572, 1620 (O-H). UV-vis (H_2_O; λ_max_, nm (ε, M^−1^cm^−1^)): 454 (973), 482 (1056), 804 (255). Elemental analysis: Anal. Calcd. for C_6_N_6_Re_3.2_Mo_2.8_S_8_ (%): C, 4.89; N, 5.71; H, 0; S, 17.42. Found: C, 4.96; N, 5.82; H, 0.14; S, 17.28. PXRD pattern shows that compound **2** is isostructural to the CaK_4_[Re_3_Mo_3_S_8_(CN)_6_]*·*8H_2_O phase reported previously [23] and does not present additional diffraction peaks.

*Preparation of (Ph_4_P)_4_[Re_3.2_Mo_2.8_S_8_(CN)_6_]* (**3**). Compound **2** (0.10 g, 0.03 mmol) was dissolved in 10 mL of H_2_O. Ph_4_PCl (0.10 g, 0.26 mmol) was dissolved in H_2_O (10 mL) and added to the solution of compound **2,** leading to the precipitation of compound **3**. The precipitate was separated by centrifugation, washed with H_2_O and dried in air. Single crystals for X-ray diffraction studies were obtained by slow diffusion of H_2_O layered under the solution of compound **3** in CH_3_CN (15 mg mL^−1^) in a thin glass tube. EDS: Re:Mo:S = 3.2:2.8:7.6. FT-IR (KBr, cm^−1^): v = 2096 (C≡N); 1436, 1107, 995, 756, 721, 686, 522 (Ph_4_P^+^). UV-vis absorption (CH_3_CN; λ_max_, nm (ε, M^−1^cm^−1^)): 475 (1238), 483 (1266), 813 (406). Elemental analysis: Anal. Calcd. for C_102_N_6_H_80_Re_3.2_Mo_2.8_S_8_ (%): C, 46.50; N, 3.19; H, 3.06; S, 9.74. Found: C, 46.43; N, 3.32; H, 3.35; S, 9.53.

*Preparation of (Bu_4_N)_4_[Re_4_Mo_2_S_8_(CN)_6_]·2H_2_O* (**4**). Compound **2** (1.00 g, 0.68 mmol) was stirred in 20 mL of H_2_O for 2 h, resulting in a dark-brown solution. The solution was filtered and added to the solution of n-Bu_4_NCl (1.00 g, 3.6 mmol) in 20 mL of H_2_O (the pH of the resulting solution was 9.6). KOH was added to the reaction mixture until it reached a pH of 11.0. The reaction mixture stayed at room temperature in the open glass and slowly evaporated. During the evaporation, a green precipitate of compound **4** was formed. The end of precipitation occurred in about 7 days. The precipitate was filtered, washed with H_2_O and dried in air. Yield: 0.52 g (33% based on initial amount of compound **2**). EDS: Re:Mo:S = 4.0:2.0:7.6. FT-IR (KBr, cm^−1^): 2115 (C≡N). UV–vis absorption (H_2_O; λ_max_, nm (ε, M^−1^cm^−1^)): 495 (425), 452 (375), 807 (140). Elemental analysis: Anal. Calcd. for C_70_N_10_H_148_O_2_Re_4_Mo_2_S_8_ (%): C, 35.7; N, 5.9; H, 6.3; S, 10.9. Found: C, 34.3; N, 5.6; H, 6.1; S, 10.9. ESI-MS in CH_3_CN, negative ion mode, *m*/*z* (Figure 2): H_4_[{Re_4_Mo_2_S_8_}(CN)_6_](H_2_O)(CH_3_CN)^2−^ (obs. 706.24, calcd. 706.24), (Bu_4_N)[{Re_4_Mo_2_S_8_}(CN)_5_]^2−^ (obs. 782.85, calcd. 782.85), (Bu_4_N)[{Re_4_Mo_2_S_8_}(CN)_4_(OH)](CH_3_CN)^2−^ (obs. 798.84, calcd. 798.86) and (Bu_4_N)_2_[{Re_4_Mo_2_S_8_}(CN)_6_]^2−^ (obs. 916.99, calcd. 916.99). PXRD pattern of polycrystalline phase matched the calculated pattern from single-crystal X-ray diffraction data. Single crystals for X-ray diffraction studies were obtained by recrystallization of **4** from CH_3_CN.

*Preparation of K_5_[Re_3_Mo_3_S_8_(CN)_6_]·8H_2_O* (**5**). The aqueous solution formed after separation of solid compound **4** was extracted with 40 mL of CH_2_Cl_2_. The organic layer was separated and evaporated in air to dryness, and the solid residue was dissolved in 15 mL of CH_3_CN. This solution was mixed with a solution of 0.30 g (3.1 mmol) of KSCN in 15 mL of CH_3_CN. A brown precipitate quickly formed after mixing the two solutions. The precipitate was dissolved in H_2_O, and KCN was added to the solution (0.01 g, 0.15 mmol). The solution was evaporated to 5 mL, which led to the precipitation of red-brown crystals. Yield: 0.17 g (25% based on initial amount of compound **2**). Elemental analysis: Anal. Calcd. for C_6_N_6_H_16_O_8_K_5_Re_3_Mo_3_S_8_ (%): C, 4.48; N, 5.23; H, 1.00; S, 15.93. Found: C, 4.71; N, 5.53; H, 0.89; S, 16.14. FT-IR (KBr, cm^−1^): 2102 (C≡N). UV–vis absorption (H_2_O; λ_max_, nm (ε, M^−1^cm^−1^)): 480 (1736), 739 (565).

*Preparation of (Ph_4_P)_5_[Re_3_Mo_3_S_8_(CN)_6_]* (**6**). A total of 300 mg (0.2 mmol) of compound **5** was dissolved in 20 mL of water. Aqueous solution of Ph_4_PCl (0.30 g, 0.8 mmol) was added, causing the formation of a brown-red precipitate. The precipitate was separated by centrifugation, washed with H_2_O and dried in air. EDS: Re:Mo:S = 2.9:3.1:8.2. FT-IR (KBr, cm^−1^): v = 2052 (C≡N); 1435, 1107, 995, 750, 721, 686, 522 (Ph_4_P^+^). Elemental analysis: Anal. Calcd. for C_126_N_6_H_100_P_5_Re_3_Mo_3_S_8_ (%): C, 51.04; N, 2.83; H, 3.40; S, 8.63. Found: C, 51.23; N, 2.78; H, 3.56; S, 8.49. ESI-MS in CH_3_CN, negative ion mode, *m*/*z* (Figure 3): (Ph_4_P)H_3_[{Re_3_Mo_3_S_8_}(CN)_5_]·(CH_3_CN)^2−^ (obs. 808.31, calcd. 808.27), (Ph_4_P)H_4_[{Re_3_Mo_3_S_8_}(CN)_4_]·(CH_3_CN)_2_^2−^ (obs. 816.31, calcd. 816.29), (Ph_4_P)H_2_[{Re_3_Mo_3_S_8_}(CN)_4_]·(CH_3_CN)_2_(H_2_O)^2−^ (obs. 824.30, calcd. 824.29), (Ph_4_P)[{Re_3_Mo_3_S_8_}(CN)_6_]·(CH_3_CN)_2_^2−^ (obs. 840.29, calcd. 840.28), (Ph_4_P)H[{Re_3_Mo_3_S_8_}(CN)_5_]·(CH_3_CN)_3_^2−^ (obs. 848.29, calcd. 848.29), (Ph_4_P)[{Re_3_Mo_3_S_8_(CN)_6_]·(CH_3_CN)_2_(H_2_O)_2_^2−^ (obs. 858.29, calcd. 858.29) and (Ph_4_P)[Re_3_Mo_3_S_8_(CN)_6_]·(CH_3_CN)_2_(H_2_O)_3_^2−^ (obs. 867.29, calcd. 867.29).

### 3.3. Single-Crystal Diffraction Studies

Single-crystal X-ray diffraction data were collected at room temperature on an APEX II Bruker AXS diffractometer using a Mo-Kα X-ray wavelength (λ = 0.71073 Å) and processed with the APEX 2 program suite [29]. Frame integration and data reduction were carried out with the program SAINT [30]. The program SADABS [31] was employed for multi-scan absorption corrections. The structures of compounds **4** and **5** were solved by direct methods using the SHELXT program [32] and refined with full-matrix least-squares methods based on F^2^ (SHELXL) [33] with the aid of the WinGX platform [34]. All non-hydrogen atoms were refined with anisotropic atomic displacement parameters. The statistical distribution of rhenium and molybdenum atoms on their respective crystallographic sites was considered by constraining equivalent atomic coordinates and anisotropic atomic displacement parameters. From preliminary refinements, a Re/Mo ratio close to 4/2 was refined for **4** and close to 3/3 for **5**. Considering that these ratios are in agreement with elemental compositions determined from experimental chemical analyses, final refinements were conducted by using the SUMP command, restraining a Re/Mo ratio of 4/2 for **4** and of 3/3 for **5**. This restriction has no influence on the reliability factors or the largest difference in peak and hole values. Hydrogen atoms were included in the structural model considering their calculated positions, and their equivalent isotropic displacement parameters were constrained to be equal to 1.5 times that of the linked atom for methyl hydrogens and 1.2 times for others. Hydrogen atoms in the water molecules were not localized. Publishing data were computed using CRYSCALC program [35]. Selected crystal, collection and refinement data for **4** and **5** are gathered in Table 6. Selected bond distances are listed in Table 2. Crystallographic data for the structures of the title compounds have been deposited at the Cambridge Crystallographic Data Center under reference numbers CCDC 2267663 and 2267664. Copies of this information may be obtained free of charge from the CCDC, 12 Union Road, Cambridge CB2 1 EZ, UK (Fax: +44-1223-336033; http://www.ccdc.cam.ac.uk/conts/retrieving.html; accessed on 6 June 2023).

### 3.4. Computational Details

Density functional theory (DFT) calculations for *cis-* and *trans-*[Re_4_Mo_2_Se_8_(CN)_6_]^n−^ cluster anions (n = 4–6) and *gran-* and *mer-*[Re_3_Mo_3_Se_8_(CN)_6_]^n−^ cluster anions (n = 5–7) were carried out in the ADF2021 program package [36,37]. Geometric parameters were optimized with the PW92+revPBE density functional [38,39] and all-electron TZ2P basis set [40]. The zero-order regular approximation (ZORA) [41] for the scalar relativistic effects and the Conductor-like Screening Model (COSMO) [42] for water environment were used in all calculations. Spin-unrestricted calculations were used for *cis-* and *trans-*[Re_4_Mo_2_Se_8_(CN)_6_]^5−^ and *gran-* and *mer-*[Re_3_Mo_3_Se_8_(CN)_6_]^6−^ cluster anions containing 23 CSEs (one unpaired electron). Calculations were performed using C_1_ symmetry. In order to facilitate comparisons with similar works in the literature, MO units are a.u. instead of e^1/2^·Å^−3/2^, which is common practice in coordination chemistry [43,44].

### 3.5. EXAFS

X-ray absorption spectroscopy (XAS) measurements were carried out at room temperature in transmission mode at the K edge of Mo and L_3_-absorption edge of Re at the beamline SAMBA [45] in the Soleil Synchrotron, France (proposal 20210623). Radiation coming from a bending magnet source was monochromatized by a Si(220) fixed-exit sagittally focusing double-crystal monochromator. Harmonic rejection was performed using a couple of mirrors that also focused the monochromatic beam vertically (spot area around 300 × 300 μm^2^). The samples were examined as self-supporting pellets (matrix cellulose), for which the amount of sample was optimized in order to have a proper XAS signal. The extended X-ray absorption fine structure (EXAFS) signal treatment was performed according to standard procedures: subtraction of the pre-edge and post-edge backgrounds, edge normalization and extraction of EXAFS signal χ(k) and its Fourier transformation, which provides a map in the real space of the distribution of the distances *R* around the absorber atom. The Demeter software package was used to perform data treatment and fitting [46] using the phase and amplitude calculated using the FEFF-10lite code [47]. Structure models for the {Re_3_Mo_3_S_8_} and {Re_4_Mo_2_S_8_} cores were obtained from crystallographic data. The occupancies of the metal positions were changed so that the rhenium and molybdenum atoms were in different positions, corresponding to the isomerism of the core (Figures S1 and S4 in Supplementary Materials). EXAFS measurements and Fourier transform magnitudes for samples **4** and **5** are given in Figures S2, S3, S5 and S6 in the Supplementary Materials, respectively. The final refinement parameters are given in Tables S1–S4 in the Supplementary Materials.

## 4. Conclusions

We have demonstrated the possibility of separating anionic complexes based on {Re_4_Mo_2_S_8_} and {Re_3_Mo_3_S_8_} heterometallic clusters and their isolation as individual compounds. These clusters are obtained from the K_6_[{Re_3.2_Mo_2.8_S_8_}(CN)_5_] polymer synthesized at high temperature. The electrochemical behavior of the [Re_3_Mo_3_S_8_(CN)_6_]^5−^ and [Re_4_Mo_2_S_8_(CN)_6_]^4−^ cluster-based anionic units is different, which makes possible the selective oxidation of the {Re_4_Mo_2_S_8_} cluster with atmospheric oxygen in an aqueous solution and the isolation of its insoluble form (Bu_4_N)_4_[{Re_4_Mo_2_S_8_}(CN)_6_]. A comparison of the properties of anionic complexes based on the {Re_4_Mo_2_S_8_}, {Re_3_Mo_3_S_8_}, {Re_4_Mo_2_Se_8_} and {Re_3_Mo_3_Se_8_} clusters shows that the charge states and spectroscopic properties of heterometallic clusters are determined by the ratio of rhenium and molybdenum atoms in the core to a much greater extent than by the type of internal chalcogenide ligands. Such anionic complexes could be further used as building blocks for the design of photoelectrodes for solar cells. Homometallic clusters have already been shown to be promising solar cell components. For example, FTO film coated by the Cs_2_[{Mo_6_I_8_}I_6_] complex displayed extremely rare ambipolar semiconducting properties, i.e., the ability to generate both holes and electrons as main charge carriers [8]. According to the published data, molybdenum cluster complexes have an energy gap of 1.9 eV, which is close to the values for heterometallic clusters—2.2 eV for {Re_3_Mo_3_S_8_} and 2.0 eV for {Re_4_Mo_2_S_8_}. Materials based on (Bu_4_N)_3_[{Re_6_Q_8_}Cl_6_] (Q = S, Se) cluster complexes also exhibit ambipolar properties [9]. The energy gaps for these clusters are 2.1 and 1.9 eV for Q = S and Se, respectively. The deposition of the clusters on the FTO surface has little effect on the energy gaps. According to the published data, we assume that heterometallic cluster complexes have similar semiconductor properties but may have different optical properties in comparison with homometallic clusters.

## Figures and Tables

**Table 1:** Correlation between CSE number and charge (n) of the [Re_4_Mo_2_Se_8_(CN)_6_]^n^ and [Re_3_Mo_3_S_8_(CN)_6_]^n^ cluster anions.

Anion/Charge	7−	6−	5−	4−
[Re_4_Mo_2_Se_8_(CN)_6_]^n^	25	24	23	22
[Re_3_Mo_3_S_8_(CN)_6_]^n^	24	23	22	21

**Table 2:** Mean bond lengths from single-crystal XRD data for Re/Mo cyanide octahedral cluster complexes.

	M–M	M–Q	M–C	Ref.
[Re_4_Mo_2_S_8_(CN)_6_]^4−^ (296 K)	2.615(1)	2.427(2)	2.15(1)	This work
[Re_4_Mo_2_Se_8_(CN)_6_]^4−^ (150 K)	2.646(14)	2.540(9)	2.16(1)	[24]
[Re_3_Mo_3_S_8_(CN)_6_]^5−^ (296 K)	2.623(2)	2.430(4)	2.15(3)	This work
[Re_3_Mo_3_Se_8_(CN)_6_]^5−^ (150 K)	2.6494(9)	2.5464(9)	2.14(2)	[24]

**Table 3:** Redox potentials (E_1/2_) for {M_6_Q_8_}-type cyanide clusters (M = Re, Mo; Q = S, Se; potentials are normalized vs. Ag/AgCl/3.5M KCl electrode).

Anion/CSE Number	21/22	22/23	23/24
[Re_6_S_8_(CN)_6_]^(n−1)−/n−^ (CH_3_CN) [27]	-	-	+0.60
[Re_4_Mo_2_S_8_(CN)_6_]^(n−1)−/n−^ (CH_3_CN)	-	−0.303	−1.186
[Re_4_Mo_2_Se_8_(CN)_6_]^(n−1)−/n−^ (CH_3_CN) [24]	-	−0.476	−1.294
[Re_3_Mo_3_S_8_(CN)_6_]^(n−1)−/n−^ (Acetone)	−0.018	−0.904	−1.332
[Re_3_Mo_3_Se_8_(CN)_6_]^(n−1)−/n−^ (CH_3_CN) [24]	−0.202	−0.870	−1.270

**Table 4:** M-M distances (Å) in [Re_3_Mo_3_Se_8_(CN)_6_]^5−^ by EXAFS, DFT and SC XRD.

	*fac-*[Re_3_Mo_3_S_8_(CN)_6_]^5−^		*mer-*[Re_3_Mo_3_S_8_(CN)_6_]^5−^		SC XRD
	EXAFS	DFT	EXAFS	DFT	
*R_Mo-Mo_*	2.75(2)	<2.695>	2.741(3)	<2.743>	
*R_Mo-Re_*	2.630(6)	<2.658>	2.642(5)	<2.649>	
*R_Re-Re_*	2.608(4)	<2.633>	2.612(6)	<2.611>	
*R_M-M_*	<2.655>	<2.661>	<2.659>	<2.659>	2.623(2)

**Table 5:** M-M distances (Å) in [Re_4_Mo_2_Se_8_(CN)_6_]^4−^ by EXAFS, DFT and SC XRD.

	*cis-*[Re_4_Mo_2_S_8_(CN)_6_]^4−^		*trans-*[Re_4_Mo_2_S_8_(CN)_6_]^4−^		SC XRD
	EXAFS	DFT	EXAFS	DFT	
*R_Mo-Mo_*	2.80(3)	<2.800>	–	–	
*R_Mo-Re_*	2.644(3)	<2.650>	2.644(3)	<2.675>	
*R_Re-Re_*	2.593(4)	<2.618>	2.592(4)	<2.589>	
*R_M-M_*	<2.635>	<2.649>	<2.627>	<2.646>	2.597(1)–2.623(1) <2.615>

**Table 6:** Summary of single-crystal data collections and structure refinement conditions of (Bu_4_N)_4_[Re_4_Mo_2_S_8_(CN)_6_]·2H_2_O and K_5_[Re_3_Mo_3_S_8_(CN)_6_]·8H_2_O.

	4	5
Empirical formula	C_70_H_148_Mo_2.13_N_10_O_2_Re_3.87_S_8_	C_6_H_16_K_5_Mo_3.06_N_6_O_8_Re_2.94_S_8_
Formula weight	2343.31	1593.39
Temperature (K)	296(2)	296(2)
Crystal size (mm^3^)	0.17 × 0.12 × 0.07	0.06 × 0.05 × 0.03
Crystal system	Monoclinic	Cubic
Space group	*P*2_1_/*c*	*Fm͞*3*m*
*Z*	2	4
Unit-cell dimensions		
*a* (Å)	13.784(2)	15.296(3)
*b* (Å)	17.069(3)	15.296(3)
*c* (Å)	20.812(4)	15.296(3)
*α* (°)	90	90
*β* (°)	102.107(5)	90
*γ* (°)	90	90
Volume (Å^3^)	4787.7(14)	3579(2)
D_calcd._ (g∙cm^−3^)	1.625	2.957
μ (mm^−1^)	5.358	12.036
θ range (°)	1.925–27.547	3.768–27.386
Indices ranges	−17 ≤ *h* ≤ 17 0 ≤ *k* ≤ 22 0 ≤ *l* ≤ 26	−19 ≤ *h* ≤ 8 −19 ≤ *k* ≤ 8 −12 ≤ *l* ≤ 16
Reflections collected	10,988	2000
Unique reflections	10,988 (*R*_int_ = 0.082)	249 (*R*_int_ = 0.0630)
Observed reflections	6548 [*I* > 2σ(*I*)]	209 [*I* > 2σ(*I*)]
Parameters refined/restraints	436/96	24/0
*R*[*F*^2^ > 2σ(*F*^2^)]	*R*_1_ = 0.0429 *wR*_2_ = 0.1039	*R*_1_ = 0.0336 *wR*_2_ = 0.0953
*R*(*F*^2^) (all data)	*R*_1_ = 0.1051 *wR*_2_ = 0.1302	*R*_1_ = 0.0399 *wR*_2_ = 0.0992
Goodness-of-fit on F^2^	0.999	1.194
Δρ_max_, Δρ_min_ (e∙Å^−3^)	1.139, −1.635	1.167, −2.842

## Data Availability

Data are contained within the article and Supplementary Materials.

## Supplementary Materials

The following supporting information can be downloaded at: https://www.mdpi.com/article/10.3390/molecules28155875/s1, Figure S1: Cluster core isomerism in [Re_4_Mo_2_S_8_(CN)_6_]; Table S1. Final fitting parameters of EXAFS spectra of (Bu_4_N)_4_[Re_4_Mo_2_S_8_(CN)_6_] (**4**) with *trans-*[Re_4_Mo_2_S_8_(CN)_6_] model; Figure S2: EXAFS measurements for (Bu_4_N)_4_[Re_4_Mo_2_S_8_(CN)_6_] (**4**) (weighted by k2) fitting with *trans-*model; Table S2: Final fitting parameters of EXAFS spectra of (Bu_4_N)_4_[Re_4_Mo_2_S_8_(CN)_6_] (**4**) with *cis-*[Re_4_Mo_2_S_8_(CN)_6_] model; Figure S3: EXAFS measurements for (Bu_4_N)_4_[Re_4_Mo_2_S_8_(CN)_6_] (**4**) (weighted by k2) fitting with *cis-*model; Figure S4: Cluster core isomerism in [Re_3_Mo_3_S_8_(CN)_6_]; Table S3: Final fitting parameters of the EXAFS spectra of K_5_[Re_3_Mo_3_S_8_(CN)_6_] (**5**) with *fac-*[Re_3_Mo_3_S_8_(CN)_6_] model; Figure S5: EXAFS measurements for K_5_[Re_3_Mo_3_S_8_(CN)_6_] (**5**) (weighted by k2), fiting with *fac-*model; Table S4: Final fitting parameters of the EXAFS spectra of K_5_[Re_3_Mo_3_S_8_(CN)_6_] (**5**) with *mer-*[Re_3_Mo_3_S_8_(CN)_6_] model; Figure S6: EXAFS measurements for K_5_[Re_3_Mo_3_S_8_(CN)_6_] (**5**) (weighted by k2), fitting with *mer-*model; Figure S7: Cyclic voltammetry curve for the K_5_[Re_4_Mo_2_S_8_(CN)_6_] salt in aqueous solution vs. Ag/AgCl reference electrode.
