# Supplementary material for: From K6[Re6−xMoxS8(CN)5] Solid Solution to Individual Cluster Complexes: Separation and Investigation of [Re4Mo2S8(CN)6]n− and [Re3Mo3S8(CN)6]n− Heterometallic Clusters
Source: Molecules. 2023 Aug 4;28(15):5875. doi: 10.3390/molecules28155875 (PMC10421489; doi:10.3390/molecules28155875)
Supplement: Supplementary file 1 [file molecules-28-05875-s001.zip › molecules-2465776-supplementary.pdf]

Electronic Supporting Information

From $K_6[Re_{6-x}Mo_xS_8(CN)_5]$ Solid Solution to Individual Cluster Complexes: Separation and Investigation of $[Re_4Mo_2S_8(CN)_6]^{n-}$ and $[Re_3Mo_3S_8(CN)_6]^{n-}$ Heterometallic Clusters

Tatiana I. Lappi [1], Yakov M. Gayfulin [1], Adèle Renaud [2], Carmelo Prestipino [2], Pierric Lemoine [3], Vadim V. Yanshole [4], Viktoria K. Muravieva [1], Stéphane Cordier [2] and Nikolai G. Naumov [1,*]

[1] Nikolaev Institute of Inorganic Chemistry SB RAS, 3, Acad. Lavrentiev Ave., 630090 Novosibirsk, Russia; tanya.lappi2@gmail.com (T.I.L.); gayfulin@niic.nsc.ru (Y.M.G.); muravyeva@niic.nsc.ru (V.K.M.)

[2] UFR Sciences et Propriétés de la Matière, Université de Rennes, CNRS, Institut des Sciences Chimiques de Rennes (ISCR) UMR 6226, F-35000 Rennes, France; adele.renaud@univ-rennes1.fr (A.R.); carmelo.prestipino@univ-rennes1.fr (C.P.); stephane.cordier@univ-rennes1.fr (S.C.)

[3] Institut Jean Lamour, UMR 7198 CNRS, Universite de Lorraine, F-54011 Nancy, France; pierric.lemoine@univ-lorraine.fr

[4] International Tomography Center SB RAS, 3A, Institutskaya Str., 630090 Novosibirsk, Russia; vadim.yanshole@tomo.nsc.ru

* Correspondence: naumov@niic.nsc.ru

EXAFS measurements and data fitting

Both data for Re-L3 edge and Mo K-edge has been fitted using a single E0 for each edge, and constrain the Mo–C and Re–C path to have the same Debye-Waller factor. The others constrain are induced by identity the Mo–Re distance is the same of Re–Mo.

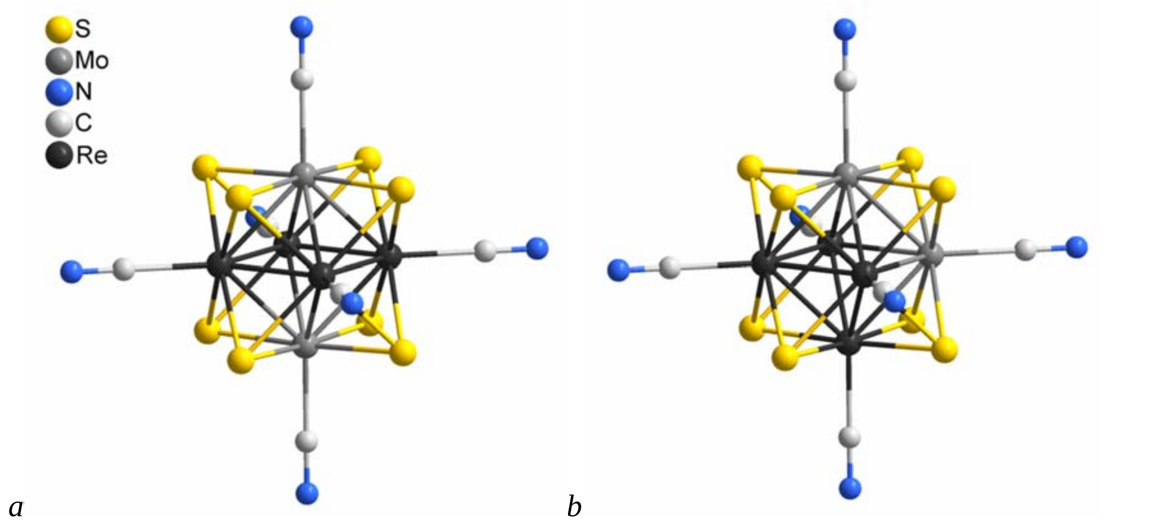

Figure S1. Cluster core isomerism in $[Re_4Mo_2S_8(CN)_6]$: a) *trans*-$[Re_4Mo_2S_8(CN)_6]$ b) *cis*-$[Re_4Mo_2S_8(CN)_6]$ models used for data fitting

Table S1. Final fitting parameters of EXAFS spectra of $(Bu_4N)_4[Re_4Mo_2S_8(CN)_6]$ (**4**) with *trans*-$[Re_4Mo_2S_8(CN)_6]$ model.

Mo K edge : k-range=3-19 R-range= 1–3 weight = 1,2,3 R-factor k^2=0.00760 S_0^2 = 1.01(7)				
	N	R	σ^2	e_0
C	1	2.18(3)	0.0005(2)	4.2(1.1)
S	4	2.420(7)	0.0024(1)	4.2(1.1)
Re	4	2.644(3)	0.0024(2)	4.2(1.1)
Re L_3 edge : k-range=2.9-17.8 R-range= 1.2–3 weight = 1,2,3 R-factor k^2=0.02355 S_0^2 = 0.90(7)				
	N	R	σ^2	e_0
C	1	2.13(2)	0.0005(2)	6.8(8)
S	4	2.413(5)	0.00254(6)	6.8(8)
Mo	2	2.644(3)	0.0021(2)	6.8(8)
Re	2	2.592(4)	0.0021(3)	6.8(8)

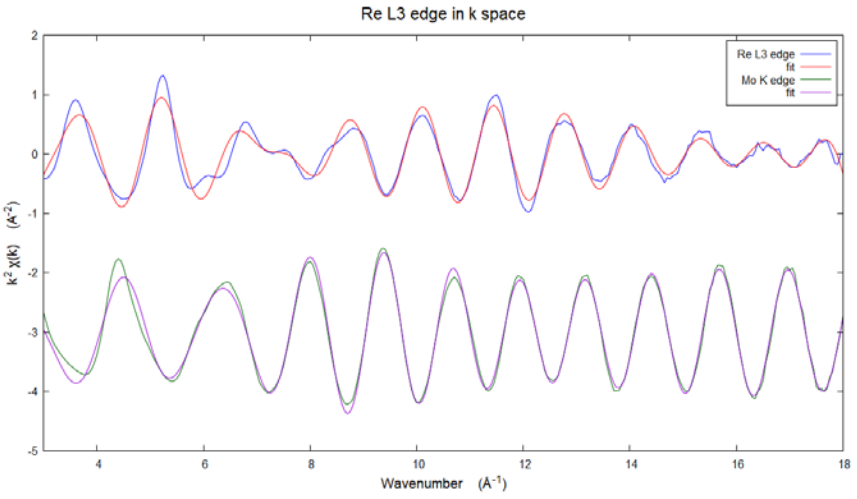

a

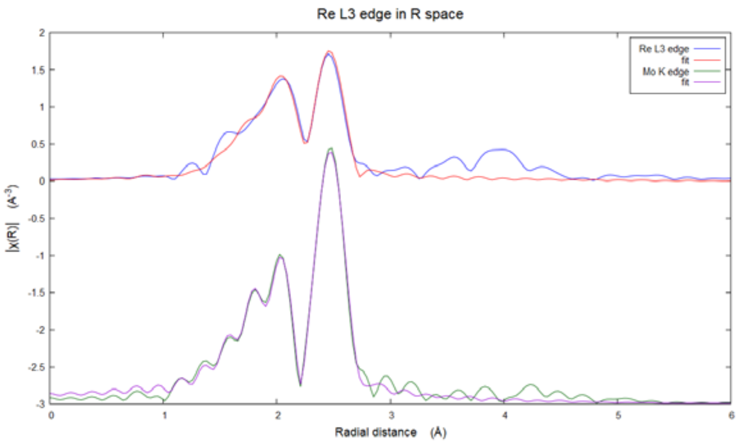

b

Figure S2. a) EXAFS measurements for $(Bu_4N)_4[Re_4Mo_2S_8(CN)_6]$ (**4**) (weighted by k^2), b) Fourier transform magnitudes. Re L3-edge experimental curves (blue), model fit with *trans*-$[Re_4Mo_2S_8(CN)_6]$ (red); Mo K-edge experimental curve (green), model fit with *trans*-$[Re_4Mo_2S_8(CN)_6]$ (purple).

Table S2. Final fitting parameters of EXAFS spectra of $(Bu_4N)_4[Re_4Mo_2S_8(CN)_6]$ (**4**) with *cis*- $[Re_4Mo_2S_8(CN)_6]$ model.

Mo K edge : k-range=3-19 R-range= 1–3 weight = 1,2,3 R-factor k^2=0.00531<br>S_0^2 = 094(7)				
	N	R	σ^2	e_0
C	1	2.13(3)	0.001(2)	4.6(1.0)
S	4	2.419(5)	0.0026(5)	4.6(1.0)
Re	3	2.644(3)	0.0017(2)	4.6(1.0)
Mo	1	2.80(3)	0.005(3)	4.6(1.0)

Re L_3 edge : k-range=2.9-17.8 R-range= 1.2–3 weight = 1,2,3 R-factor k^2=0.02759<br>S_0^2 = 0.90(7)				
	N	R	σ^2	e_0
C	1	2.20(5)	0.001(2)	8.8(8)
S	4	2.413(5)	0.00254(6)	8.8(8)
Mo	2	2.644(3)	0.0017(2)	8.8(8)
Re	2	2.593(4)	0.0027(4)	8.8(8)

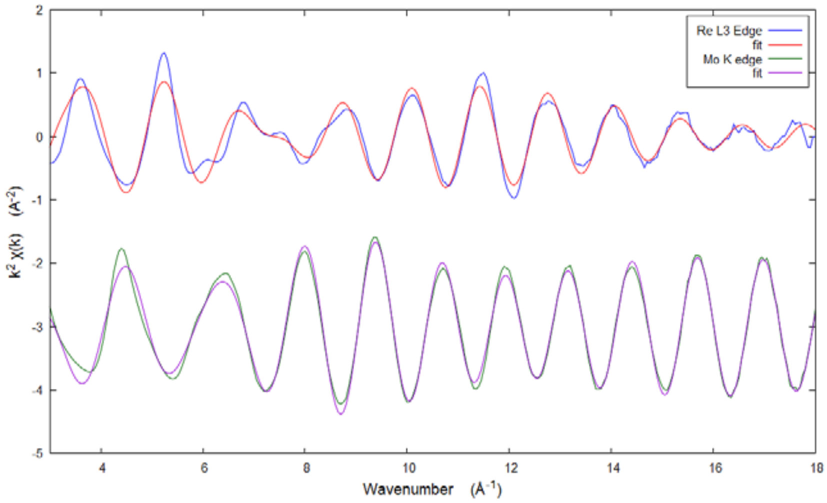

a

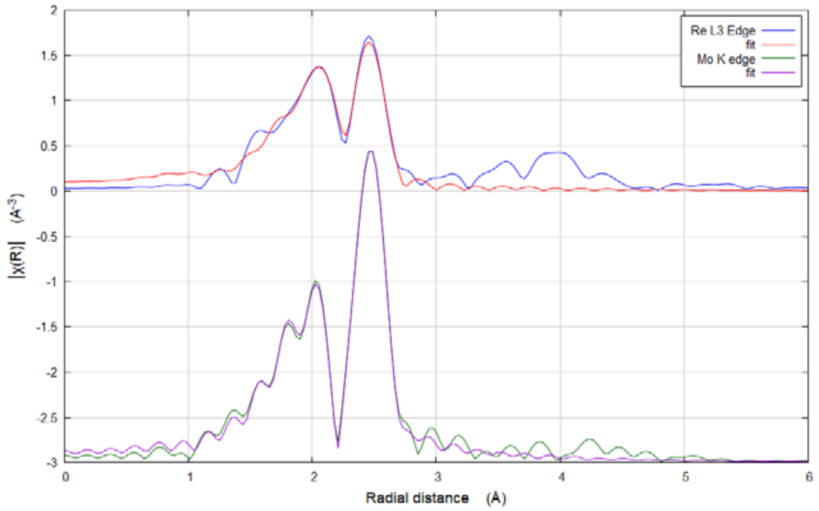

b

Figure S3. a) EXAFS measurements for $(Bu_4N)_4[Re_4Mo_2S_8(CN)_6]$ (**4**) (weighted by k^2), b) Fourier transform magnitudes. Re L3-edge experimental curves (blue), model fit with *cis*-$[Re_4Mo_2S_8(CN)_6]$ (red); Mo K-edge experimental curve (green), model fit with *cis*-$[Re_4Mo_2S_8(CN)_6]$ (purple).

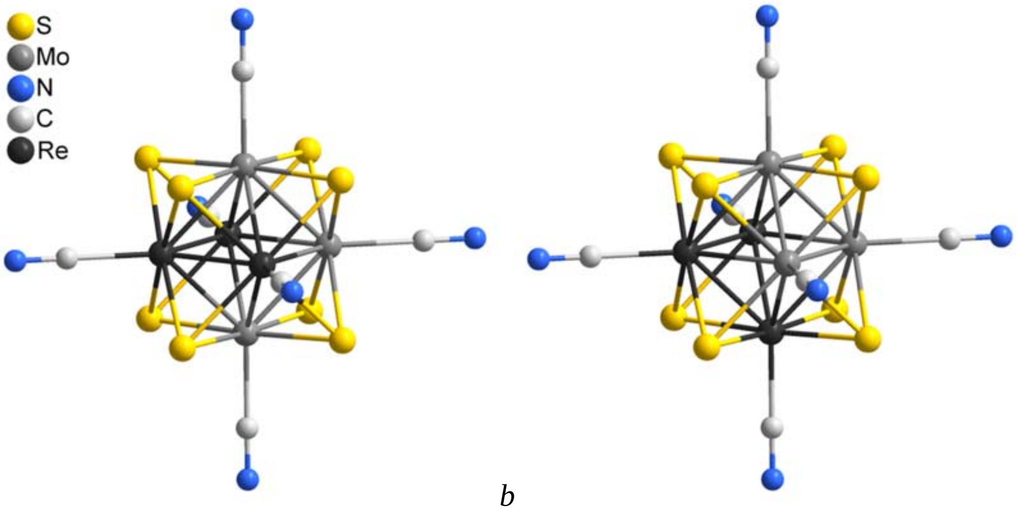

a *b*

Figure S4. Cluster core isomerism in $[Re_3Mo_3S_8(CN)_6]$: a) *mer*-$[Re_3Mo_3S_8(CN)_6]$ b) *fac*-$[Re_3Mo_3S_8(CN)_6]$ models used for data fitting.

Table S3. Final fitting parameters of the EXAFS spectra of $K_5[Re_3Mo_3S_8(CN)_6]$ (**5**) with *fac*-$[Re_3Mo_3S_8(CN)_6]$ model.

Mo K edge : k-range=3-19 R-range= 1–3 weight = 1,2,3 R-factor k^2=0.00760<br>S_0^2 = 0.91 (1)				
	N	R	σ^2	e_0
C	1	2.17(7)	0.00100	0.8(9)
S	4	2.45(1)	0.0036(1)	0.8(9)
Mo	2	2.75(2)	0.0026(8)	0.8(9)
Re	2	2.630(6)	0.0020(5)	0.8(9)
Re L_3 edge : k-range=2.9-17.8 R-range= 1.2–3 weight = 1,2,3 R-factor k^2=0.02355<br>S_0^2 = 0.88(6)				
	N	R	σ^2	e_0
C	1	2.11(3)	0.00100	5.8(9)
S	4	2.407(6)	0.0023(5)	5.8(9)
Mo	2	2.630(6)	0.0020(5)	5.8(9)
Re	2	2.608(4)	0.0015(5)	5.8(9)

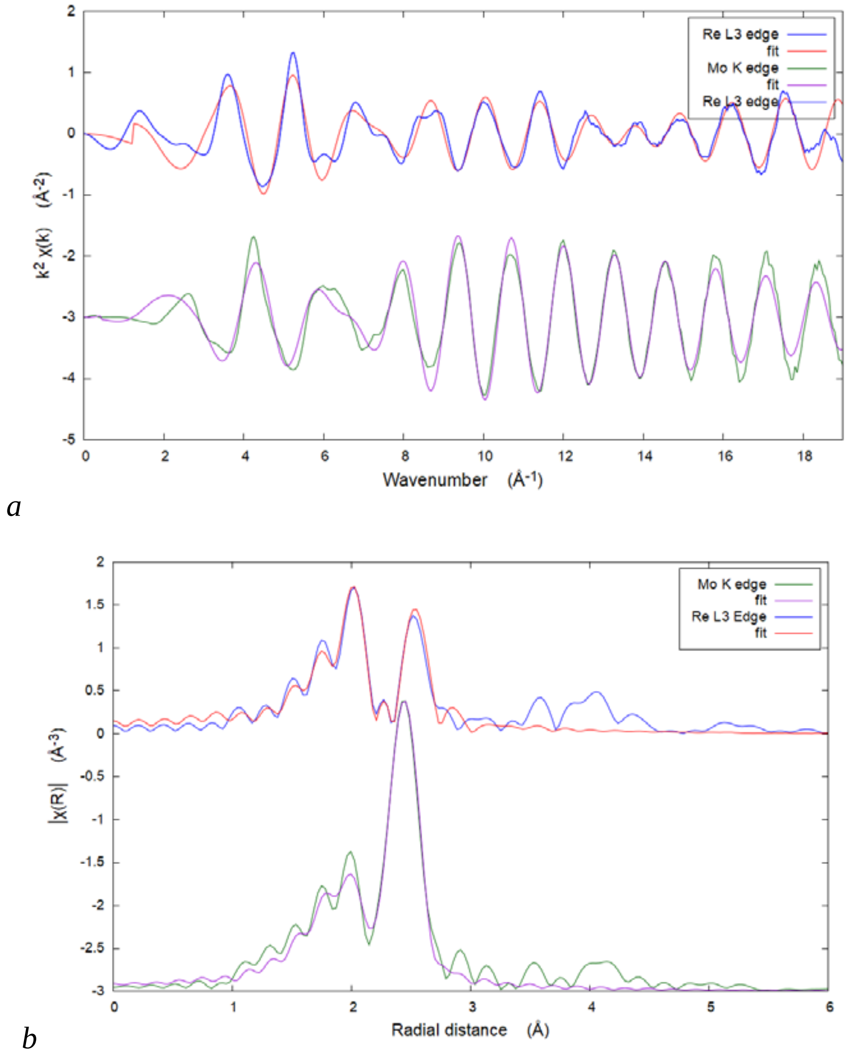

Figure S5. a) EXAFS measurements for $K_5[Re_3Mo_3S_8(CN)_6]$ (**5**) (weighted by k^2), b) Fourier transform magnitudes. Re L3-edge experimental curves (blue), model fit with *fac*-$[Re_3Mo_3S_8(CN)_6]$ (red); Mo K-edge experimental curve (green), model fit with *fac*-$[Re_3Mo_3S_8(CN)_6]$ (purple).

Table S4. Final fitting parameters of the EXAFS spectra of $K_5[Re_3Mo_3S_8(CN)_6]$ (**5**) with *mer*-$[Re_3Mo_3S_8(CN)_6]$ model.

Mo K edge : k-range=3-19 R-range= 1–3 weight = 1,2,3 R-factor k^2=0.00760<br>S_0^2 = 1.33 (1)				
	N	R	σ^2	e_0
C	1	2.13(5)	0.001	0.08(9)
S	4	2.457(8)	0.0062(5)	0.08(9)
Re	2.667	2.642(5)	0.0042(7)	0.08(9)
Mo	1.333	2.741(3)	0.0010(8)	0.08(9)
Re L_3 edge : k-range=2.9-17.8 R-range= 1.2–3 weight = 1,2,3 R-factor k^2=0.02355<br>S_0^2 = 0.88(6)				
	N	R	σ^2	e_0
C	1	2.12(3)	0.001	6.8(9)
S	4	2.41(1)	0.0021(6)	6.8(9)
Mo	2.667	2.642(5)	0.0042(5)	6.8(9)
Re	1.333	2.612(6)	0.0010(7)	6.8(9)

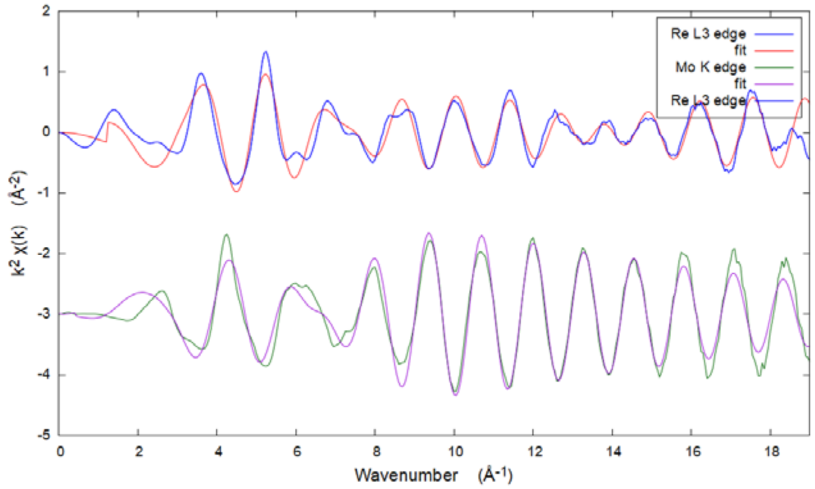

a

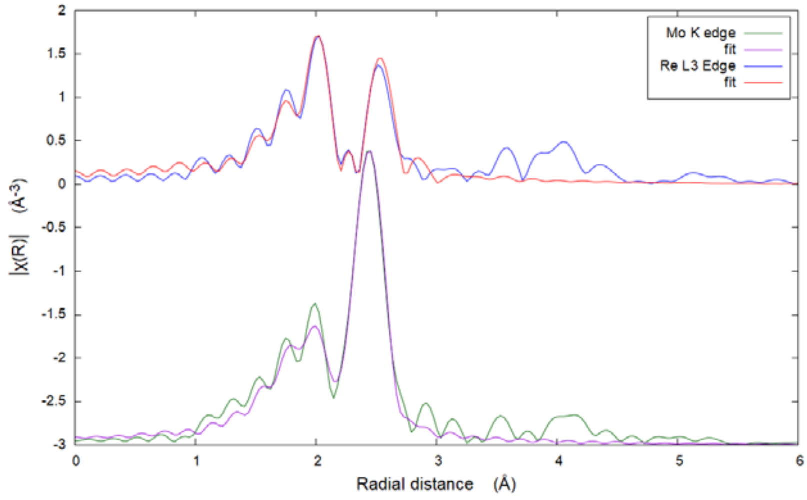

b

Figure S6. a) EXAFS measurements for $K_5[Re_3Mo_3S_8(CN)_6]$ (**5**) (weighted by k^2), b) Fourier transform magnitudes. Re L3-edge experimental curves (blue), model fit with *mer*-$[Re_3Mo_3S_8(CN)_6]$ (red); Mo K-edge experimental curve (green), model fit with *mer*-$[Re_3Mo_3S_8(CN)_6]$ (purple).

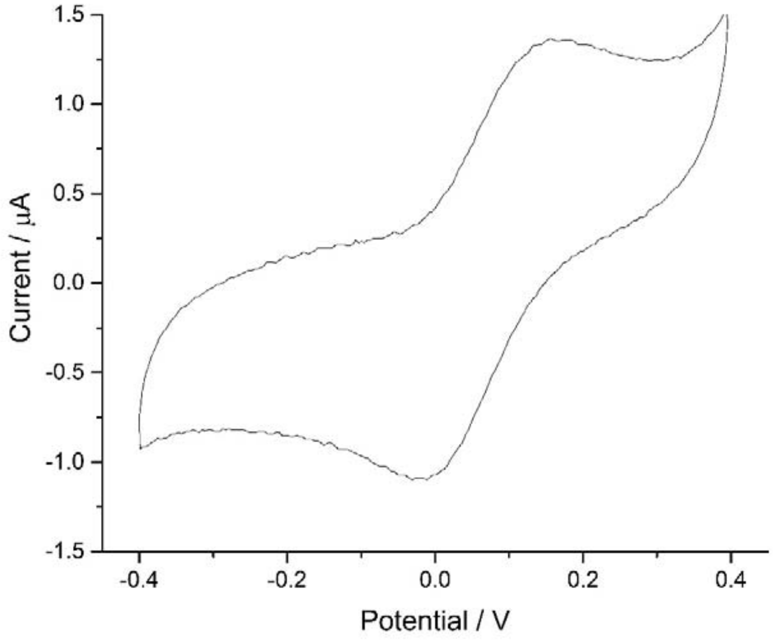

Figure S7. Cyclic voltammetry curve for the $K_5[Re_4Mo_2S_8(CN)_6]$ salt in aqueous solution vs Ag/AgCl reference electrode. Scan rate was 50 mV·s^{-1}.
